# New-Onset Pemphigus Following Drug Exposure and Vaccination: A Systematic Review of Reported Cases

**DOI:** 10.3390/vaccines14090828

**Published:** 2026-09-20

**Authors:** Gabriele Poddine, Francesco Bellinato, Paolo Gisondi, Giampiero Girolomoni

**Affiliations:** Section of Dermatology and Venereology, Department of Medicine, University of Verona, Piazzale Stefani, 1, 37126 Verona, Italy; gabriele.poddine@studenti.univr.it (G.P.); paolo.gisondi@univr.it (P.G.); giampiero.girolomoni@univr.it (G.G.)

**Keywords:** pemphigus, drug-associated pemphigus, vaccine-associated pemphigus, autoimmune blistering diseases, vaccination, pharmacovigilance, systematic review

## Abstract

Background: Although an increasing number of cases of new-onset pemphigus have been reported following drug exposure or vaccination, the available evidence remains fragmented and distinguishing true trigger-associated disease from coincidental onset continues to represent a major clinical challenge. We performed a systematic review to evaluate the available evidence on drug- and vaccine-associated new-onset pemphigus and to compare their clinical characteristics, management, and outcomes. Methods: This systematic review was conducted according to the PRISMA 2020 statement and prospectively registered in PROSPERO (CRD420261307622). PubMed was searched from database inception to 30 April 2026. Studies reporting individual patients with new-onset pemphigus temporally associated with drug exposure or vaccination were included. Demographic, clinical, immunopathological, therapeutic, and outcome data were extracted and synthesized descriptively because of the anticipated heterogeneity of the available evidence. Results: A total of 20 drug-associated and 26 vaccine-associated cases identified from primary reports were included in the descriptive synthesis. Drug-associated cases demonstrated marked heterogeneity in the implicated agents, broader clinical variability, and a longer median latency, with frequent clinical improvement following withdrawal of the suspected drug when reported. In contrast, vaccine-associated cases occurred predominantly after SARS-CoV-2 vaccination, displayed a substantially shorter latency, and were mainly represented by pemphigus vulgaris and pemphigus foliaceus. Across both groups, systemic corticosteroids constituted the mainstay of treatment, with generally favorable outcomes among patients with available follow-up. However, the available evidence consisted almost exclusively of case reports and small case series, precluding reliable assessment of incidence or causality. Conclusions: Current evidence suggests that drugs and vaccines may act as potential triggers of new-onset pemphigus in susceptible individuals; however, the strength of evidence differs substantially between the two settings. Drug-associated cases generally provide more convincing clinical support for a trigger-related mechanism, whereas vaccine-associated cases require more cautious interpretation because temporal association alone cannot establish causality. Standardized case reporting, prospective pharmacovigilance, and mechanistic studies are needed to strengthen causal inference and improve the recognition and management of trigger-associated pemphigus.

## 1. Introduction

Pemphigus is a group of rare, potentially life-threatening autoimmune blistering diseases characterized by the production of pathogenic autoantibodies directed against desmosomal adhesion molecules, primarily desmoglein 1 (Dsg1) and desmoglein 3 (Dsg3). Clinically, pemphigus encompasses a spectrum of entities, including pemphigus vulgaris (PV), superficial pemphigus, and less common variants such as pemphigus vegetans, IgA pemphigus, and pemphigus herpetiformis, with manifestations ranging from mucosal erosions to widespread cutaneous blistering and erosions. The diagnosis of pemphigus is suspected based on clinical manifestations and confirmed by histopathological examination, direct immunofluorescence, and the detection of tissue-bound and circulating autoantibodies against desmosomal antigens. Despite substantial advances in diagnostic techniques and therapeutic strategies, pemphigus remains associated with significant morbidity, and its pathogenesis is not yet fully understood [1,2,3].

In addition to idiopathic forms, an increasing body of evidence supports the role of external triggers in the development of pemphigus, particularly drugs and, more recently, vaccines. Drug-associated pemphigus has been recognized for decades and has been associated with a wide range of pharmacological agents, including thiol-containing drugs, angiotensin-converting enzyme inhibitors, antibiotics, and biologic or targeted therapies. These forms may present with clinical and immunopathological features overlapping those of idiopathic pemphigus, although variability in latency, severity, and response to treatment has been reported. The expanding spectrum of implicated agents highlights that trigger-associated pemphigus encompasses diverse pharmacological classes and novel immunomodulatory therapies [4,5,6,7,8].

More recently, the widespread administration of vaccines, particularly in the context of the COVID-19 pandemic, has been accompanied by an increasing number of reports describing new-onset pemphigus following vaccination. Proposed mechanisms include immune system activation, molecular mimicry, and bystander activation, although a definitive causal relationship remains difficult to establish. The clinical spectrum of vaccine-associated pemphigus appears heterogeneous, and the currently available evidence is largely limited to case reports, small case series, and systematic reviews of published cases [7,9].

A key challenge in both drug- and vaccine-associated pemphigus lies in distinguishing true trigger-induced disease from coincidental onset, given the rarity of the condition and the widespread exposure to medications and vaccines in the general population [4,7,9].

To date, no comprehensive and updated systematic review has specifically focused on new-onset pemphigus associated with both drugs and vaccines, with particular emphasis on demographic characteristics, triggering agents, latency, clinical presentation, management, and outcomes. Given the increasing use of biologic and targeted therapies, together with the global implementation of vaccination programs, an updated synthesis of the available evidence is warranted to improve recognition of potential trigger-associated pemphigus and to better inform clinical decision-making [5,9].

The primary objective of this systematic review was to analyze the available evidence on new-onset pemphigus associated with drugs and vaccines, focusing on demographic characteristics, triggering agents, latency, clinical presentation, treatment strategies, and outcomes. The secondary objective was to explore clinical and temporal patterns that may support a causal relationship between exposure and disease onset and to provide a comprehensive overview of the available evidence to facilitate clinical recognition and management.

## 2. Materials and Methods

This systematic review was conducted in accordance with the Preferred Reporting Items for Systematic Reviews and Meta-Analyses (PRISMA) 2020 statement [10]. The review protocol was prospectively registered in the International Prospective Register of Systematic Reviews (PROSPERO) (registration number: CRD420261307622).

The review aimed to identify and synthesize all available evidence regarding new-onset pemphigus occurring after exposure to drugs or vaccines. Eligible studies included patients of any age with a diagnosis of pemphigus temporally associated with a pharmacological agent or vaccination. The diagnosis of pemphigus was required to be supported by clinical, histopathological, and/or immunological findings according to current international recommendations [1]. All pemphigus subtypes were considered eligible, including pemphigus vulgaris, pemphigus foliaceus, pemphigus vegetans, pemphigus herpetiformis, IgA pemphigus, and other less common variants.

Case reports, case series, retrospective and prospective observational studies, and cohort studies were eligible for inclusion. Reviews, editorials, commentaries, conference abstracts without full text, studies lacking sufficient patient-level information, duplicate publications, and non-human studies were excluded.

A systematic literature search was performed on PubMed/MEDLINE from inception to 30 April 2026 to identify published case reports of drug- and vaccine-associated pemphigus. The search strategies executed in PubMed/MEDLINE were structured as follows: Drug-associated search string: ((Pemphigus) OR (pemphigus vulgaris)) AND ((Drug-induced) OR (drug induced) OR (iatrogenic) OR (drug-related)). Vaccine-associated search string: ((Pemphigus) OR (pemphigus vulgaris)) AND ((Vaccine) OR (vaccination)).

The electronic database search was supplemented by manual screening of reference lists from retrieved primary reports and relevant literature reviews to identify additional eligible cases. The systematic literature search was restricted to PubMed/MEDLINE and to English-language publications.

**Figure 1:**
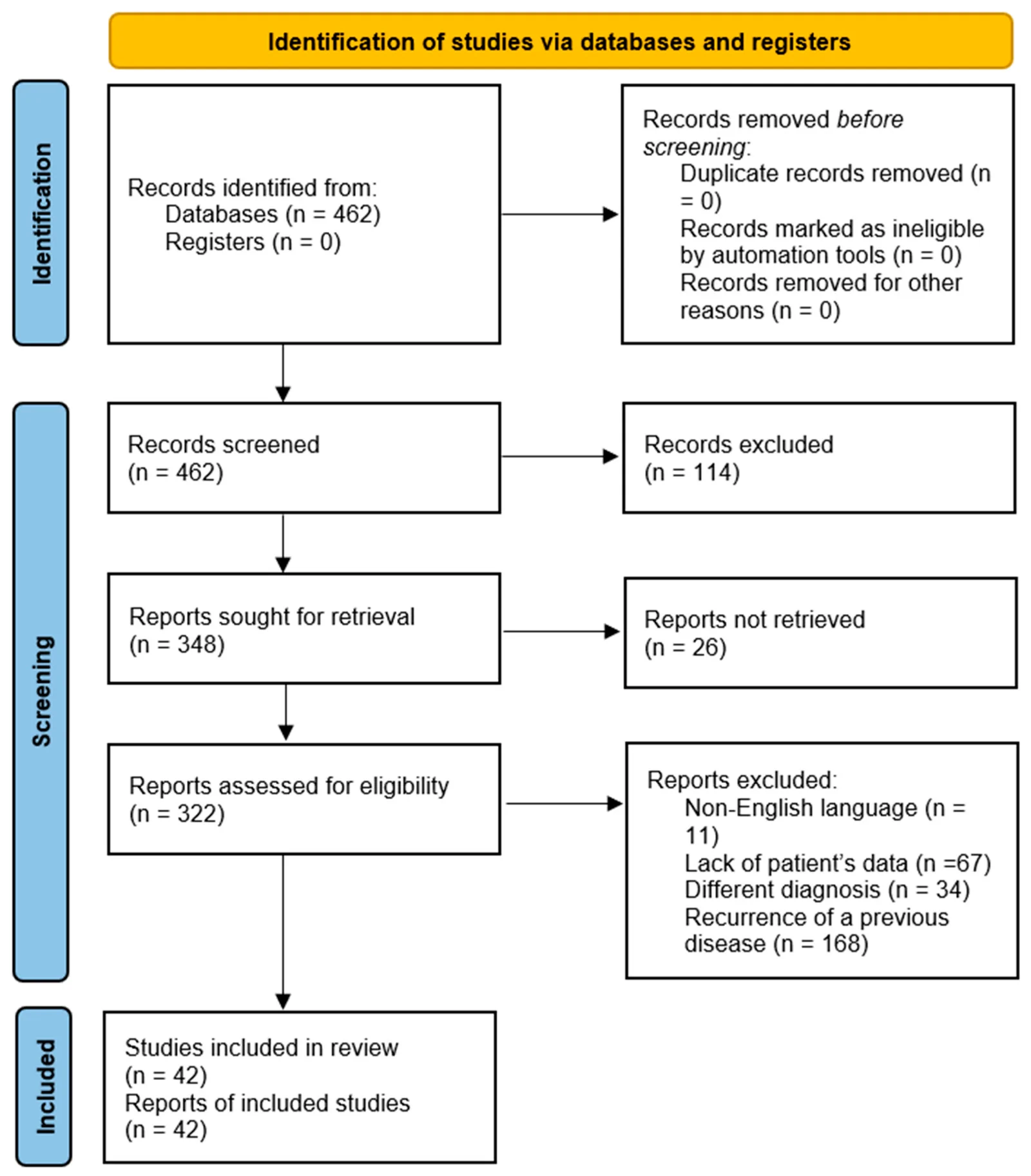
PRISMA flow diagram showing study selection process.

All retrieved records were imported into a reference management software, and duplicate records were removed prior to screening. Two investigators (G.P. and F.B.) independently screened titles and abstracts to identify potentially eligible studies. Full texts of selected articles were subsequently reviewed for inclusion. Any disagreements were resolved through discussion and consensus, with arbitration by a third senior investigator (G.G.) when necessary. This systematic review was conducted in accordance with the Preferred Reporting Items for Systematic Reviews and Meta-Analyses (PRISMA) 2020 statement (Figure 1) [10]; the completed PRISMA 2020 checklist is provided in the Supplementary Materials (Supplementary File S1).

Data extraction was independently performed by G.P. and F.B. using a standardized data collection form. Extracted variables included study characteristics (authors, year of publication, country, and study design), demographic data (age and sex), characteristics of the suspected trigger (drug, drug class, vaccine type, and latency period), clinical presentation (cutaneous, mucosal, or mucocutaneous involvement), pemphigus subtype, histopathological and immunological findings, treatment strategies, withdrawal of the suspected causative agent when applicable, clinical outcomes, time to resolution, and disease recurrence when reported.

The methodological quality of the 42 included primary publications (describing a total of 46 patients: 20 drug-associated and 26 vaccine-associated) was evaluated using the standardized Joanna Briggs Institute (JBI) critical appraisal checklist for case reports at the study level (*N* = 42). Diagnostic tests and results (Q4) were clearly described in 100% of the included studies. Because the included case series consisted of small aggregations of individual case descriptions (1–3 patients per article) without group-level statistical aggregation, the JBI 8-item checklist for case reports was applied uniformly across all publications to evaluate reporting completeness. Overall, the primary literature demonstrated high completeness of reporting across key clinical and diagnostic domains (Table 1). Demographic details (Q1) and clinical presentations (Q3) were provided in 95.2% and 97.6% of studies, respectively. Minor reporting gaps were restricted to variable detail regarding adverse events or alternative potential triggers (Q7, 78.6%). Overall, 90.5% of published reports fulfilled at least 7 of the 8 appraisal criteria, reflecting good reporting completeness in the analyzed primary literature; however, high reporting quality in case reports reflects descriptive thoroughness rather than low risk of bias for causal inference.

## 3. Drug-Associated Pemphigus

A total of 20 patients with new-onset drug-associated pemphigus were identified from primary case reports and small case series and were included in the descriptive synthesis. A previously published systematic review summarized 170 cases of drug-associated pemphigus [5]; however, these cases were not included in the present descriptive synthesis because the review represented a secondary source and individual primary reports could not be systematically distinguished from or analyzed alongside the primary studies identified by our search. The previous systematic review was therefore considered only as contextual evidence and discussed separately.

Sex was reported for 15/20 patients (75.0%), with 9 males (60.0%) and 6 females (40.0%), corresponding to a male-to-female ratio of 1.5:1. Age at onset was available for 16/20 patients (80.0%), ranging from 5 to 73 years, with a mean age of 55.4 years (SD 17.7) and a median of 60 years (interquartile range [IQR] 50.5–68.3).

Information on the implicated drug was available for all patients. The most frequently reported trigger was secukinumab, observed in 3/20 patients (15.0%). All other drugs were reported in single cases (1/20, 5.0% each), including nivolumab, tislelizumab, gefitinib, lenvatinib, liraglutide, linagliptin, lisinopril, ethambutol, piperacillin-tazobactam and/or linezolid, imiquimod, sodium valproate, Leucogen, and ibuprofen.

When grouped by pharmacological class, immune-modulating and oncologic therapies accounted for the largest proportion of cases (7/20 patients, 35.0%), followed by cardiometabolic agents (3/20, 15.0%) and anti-infective agents (2/20, 10.0%), while the remaining cases were distributed across heterogeneous drug classes.

Time to onset was reported in 11/20 patients (55.0%), ranging from 10 to 365 days, with a mean latency of 79.8 days (SD 104.8) and a median of 42 days (IQR 21–75).

Data on clinical involvement were available for 12/20 patients (60.0%). Among these, cutaneous-only involvement was observed in 7/12 patients (58.3%), whereas mucocutaneous involvement was reported in 5/12 patients (41.7%). No cases of isolated mucosal involvement were reported.

Pemphigus subtype was specified in 15/20 patients (75.0%). The most frequent phenotype was pemphigus vulgaris and related variants, observed in 5/15 patients (33.3%). Other subtypes included pemphigus herpetiformis in 2/15 patients (13.3%), and pemphigus vegetans in 2/15 (13.3%), both variants of pemphigus vulgaris. Less frequently reported were superficial variants (4/15, 26.7%), including pemphigus erythematosus (*n* = 2), pemphigus foliaceus (*n* = 1), and unspecified superficial pemphigus (*n* = 1).

Histopathological confirmation by skin biopsy was reported in all included cases, supporting the diagnosis of pemphigus. Immunological findings were inconsistently reported; when available, circulating autoantibodies against Dsg1 and/or Dsg3 were detected.

Treatment data were available for 13/20 patients (65.0%). Discontinuation of the suspected causative drug was reported in 11/13 patients (84.6%). Systemic corticosteroids were administered in 9/13 patients (69.2%), while topical corticosteroids were used in 5/13 patients (38.5%). Additional immunosuppressive or immunomodulatory therapies were reported in 4/13 patients (30.8%).

Outcome data were available for 10/20 patients (50.0%). Complete remission (CR) was achieved in 5/10 patients (50.0%), whereas partial remission (PR) was reported in the remaining 5/10 patients (50.0%), with no documented non-responders. Among patients in whom discontinuation of the suspected drug was described, clinical improvement was observed in the majority of cases.

A temporal relationship between drug exposure and disease onset was reported, and clinical improvement following drug withdrawal was observed in several patients with available follow-up; however, these observations alone do not establish a causal relationship.

Data regarding time to clinical resolution and relapse were inconsistently reported and were therefore not suitable for quantitative analysis.

## 4. Vaccine-Associated Pemphigus

A total of 26 patients with new-onset vaccine-associated pemphigus were identified across 22 primary reports. One systematic review summarized 59 cases of pemphigus following SARS-CoV-2 vaccination, including 46/59 new-onset cases (78.0%) [7]; however, this study was not pooled with the individual cases because it included both new-onset and relapsing disease and because of potential overlap with the primary reports.

Sex was reported for all 26 patients, with 15 females (57.7%) and 11 males (42.3%), corresponding to a female-to-male ratio of approximately 1.4:1. Age was available for all patients, ranging from 11 to 89 years, with a mean age of 50.2 years and a median age of 48.5 years (interquartile range [IQR] 37.0–64.8).

Information on the vaccine involved was available for all patients. COVID-19 vaccines accounted for 19/26 cases (73.1%), while non-COVID-19 vaccines were implicated in 7/26 cases (26.9%). Among COVID-19 vaccine-associated cases, mRNA vaccines were reported in 9/19 patients (47.4%), adenoviral vector vaccines in 3/19 patients (15.8%), and inactivated vaccines in 1/19 patient (5.3%). In the remaining 6/19 COVID-19 vaccine-associated cases (31.6%), the specific vaccine platform was not reported. Non-COVID-19 vaccines included diphtheria-tetanus-pertussis, tetanus-diphtheria, hepatitis B, rabies, anthrax, and influenza.

Time to onset was reported in 18/26 patients (69.2%), ranging from 1 to 60 days, with a mean latency of 17.3 days and a median latency of 10 days (IQR 7.0–20.8).

Data on clinical involvement were available for 20/26 patients (76.9%). Mucocutaneous involvement was observed in 10/20 patients (50.0%), cutaneous-only involvement in 7/20 patients (35.0%), and isolated mucosal involvement in 3/20 patients (15.0%).

Pemphigus subtype was reported for all 26 patients. Pemphigus vulgaris was the most frequent phenotype, observed in 13/26 patients (50.0%), followed by pemphigus foliaceus in 9/26 patients (34.6%), pemphigus vegetans in 2/26 patients (7.7%), and IgA pemphigus in 1/26 patient (3.8%). One case was reported as unspecified pemphigus (1/26, 3.8%).

Histopathological confirmation by skin biopsy was detailed in all the included cases, supporting the diagnosis of pemphigus.

Treatment data were available for 20/26 patients (76.9%). Systemic corticosteroids were administered in 18/20 patients (90.0%), either alone or in combination with other therapies. Topical corticosteroids or other topical treatments were used in 8/20 patients (40.0%). Additional systemic therapies included azathioprine in 5/20 patients (25.0%), rituximab in 4/20 patients (20.0%), intravenous immunoglobulins in 2/20 patients (10.0%), and methotrexate, mycophenolate mofetil, cyclosporine, or intramuscular triamcinolone in single cases. One patient was managed without systemic treatment.

Outcome data were available for 19/26 patients (73.1%). Partial remission was reported in 12/19 patients (63.2%), whereas complete remission was achieved in 7/19 patients (36.8%). No cases of non-response were documented among patients with available outcome data. Time to clinical resolution was reported in 10/26 patients (38.5%), ranging from 7 days to 6 months, with a median time to resolution of approximately 2 months.

A temporal association between vaccination and pemphigus onset was documented in the reported new-onset cases; however, causal attribution remains inherently uncertain given the lack of re-challenge and inability to evaluate de-challenge in the vaccine setting.

## 5. Discussion

This systematic review provides an updated synthesis of the available evidence on new-onset pemphigus temporally associated with drug exposure or vaccination. Overall, our findings suggest that both medications and vaccines may act as potential triggers of pemphigus in susceptible individuals; however, the current body of evidence remains largely based on isolated case reports and small case series, precluding definitive conclusions regarding causality [1,2,11]. Beyond the descriptive findings, our analysis highlights distinct clinical patterns between drug- and vaccine-associated cases. Drug-associated pemphigus was characterized by greater etiological heterogeneity, a longer latency, and frequent clinical improvement following withdrawal of the suspected agent, whereas vaccine-associated cases showed a shorter latency and were predominantly represented by pemphigus vulgaris and pemphigus foliaceus. These observations suggest that different immune perturbations may converge toward a common autoimmune phenotype in predisposed individuals, although the underlying mechanisms remain incompletely understood [3,4,5,6,7,12,13].

These differences should be interpreted as descriptive patterns rather than formal between-group comparisons, given the small number of primary cases, heterogeneity of reporting, and absence of comparative observational studies [10].

Pemphigus is a prototypical antibody-mediated autoimmune blistering disease in which pathogenic IgG autoantibodies target the desmosomal adhesion molecules desmoglein 3 (Dsg3) and desmoglein 1 (Dsg1), ultimately leading to loss of keratinocyte adhesion and intraepidermal blister formation [2,12,13]. Although idiopathic pemphigus is thought to arise from a complex interplay between genetic susceptibility (such as specific HLA class II alleles) and environmental factors, the identification of external triggers remains of considerable clinical interest [14,15,16]. This suggests that diverse exogenous stimuli, including various pharmacological agents and vaccines, may converge toward a common pathogenic pathway in susceptible individuals [8,12,13,17,18].

The mechanisms by which these exposures contribute to disease onset are likely multifactorial and only partially understood [3,4,19]. Proposed pathways include disruption of central and peripheral immune tolerance, nonspecific immune activation, epitope spreading, molecular mimicry, bystander activation, and dysregulation of autoreactive B- and T-cell responses [19,20,21,22]. In drug-associated pemphigus, both direct biochemical effects on keratinocyte adhesion and indirect immunomodulatory mechanisms have been proposed, depending on the pharmacological class involved [4,5,23]. In contrast, vaccine-associated cases are thought to reflect a transient activation of innate and adaptive immune responses capable of amplifying pre-existing autoreactivity rather than inducing de novo autoimmunity [7,9,21]. However, these hypotheses remain largely speculative, as mechanistic studies specifically addressing trigger-associated pemphigus are scarce and most published evidence relies on temporal associations rather than biological proof of causality [7,9,22].

Rather than representing distinct nosological entities, drug- and vaccine-associated pemphigus are more likely to reflect clinical scenarios in which an external immune perturbation unmasks clinically silent autoimmunity in genetically predisposed individuals [2,14,24]. This interpretation is consistent with the exceptional rarity of reported cases despite the widespread use of the implicated drugs and the global implementation of vaccination programs, suggesting that host-related susceptibility factors are likely to play a far greater role than the triggering exposure itself [2,19,25]. Future mechanistic studies integrating genetic background, autoantibody profiling, and longitudinal immune monitoring will be essential to clarify why only a small subset of exposed individuals ultimately develops disease [13,19,26].

Historically, drug-induced pemphigus has been predominantly associated with thiol-containing compounds, particularly D-penicillamine, as well as other agents including angiotensin-converting enzyme inhibitors, antibiotics, and pyrazolone derivatives [4,5,6,23]. However, the contemporary cases identified in our review illustrate a clear evolution in the spectrum of implicated medications [4,5]. Immune-modulating and oncologic therapies, including immune checkpoint inhibitors (e.g., anti-PD-1/PD-L1 agents), biologic therapies, and targeted therapies, accounted for a substantial proportion of recently reported cases [14,15,27,28,29,30]. This trend likely reflects not only the expanding clinical use of these agents but also their intrinsic capacity to perturb immune homeostasis [27,28]. By enhancing immune activation, interfering with peripheral tolerance, or selectively modulating inflammatory pathways, these therapies may facilitate the emergence of autoreactive B- and T-cell responses, thereby unmasking latent autoimmunity or promoting the development of de novo autoimmune blistering disease [28,29,30,31].

The marked heterogeneity observed among drug-associated cases further supports the concept that trigger-associated pemphigus is unlikely to arise through a single pathogenic mechanism [4,5,23]. The implicated medications belonged to diverse pharmacological classes, latency varied considerably, and multiple clinical phenotypes were observed, suggesting that different immunological pathways may converge toward a common clinical endpoint [4,23,27]. For classical thiol-containing drugs, direct biochemical interactions with keratinocyte adhesion molecules have long been proposed, whereas newer biologic and targeted therapies are more likely to induce disease through immune dysregulation and loss of self-tolerance [5,23,28]. Accordingly, the median latency of approximately six weeks observed in our analysis is more consistent with a progressive breakdown of immune tolerance than with an immediate hypersensitivity reaction [27,29,32].

An additional observation supporting a potential pathogenic role of drugs is the frequent clinical improvement following withdrawal of the suspected agent, which was documented in most patients with available follow-up [5,23]. Nevertheless, this finding should be interpreted cautiously, as concomitant immunosuppressive therapy, the absence of rechallenge in nearly all published cases, the possibility of spontaneous fluctuations in disease activity, and publication bias substantially limit causal inference [5,6,32,33]. Consequently, although the available evidence is compatible with a drug-triggered mechanism in many patients, a definitive cause-and-effect relationship cannot be established on the basis of the currently available literature [5,27,32,33].

Vaccine-associated cases displayed a distinct clinical profile compared with drug-associated pemphigus [7,9]. Most reports followed SARS-CoV-2 vaccination, an observation that is unsurprising given the unprecedented scale of global COVID-19 vaccination campaigns and the heightened pharmacovigilance efforts implemented during the pandemic [7,9,34]. However, pemphigus has also been reported following several non-COVID vaccines, including hepatitis B, influenza, tetanus-containing, rabies, and anthrax vaccines indicating that the phenomenon is not restricted to a single vaccine platform or immunization strategy [7,9,21,35].

The shorter median latency observed after vaccination (approximately 10 days in our cohort) may be more consistent with rapid activation of pre-existing autoreactive immune pathways than with the de novo generation of pathogenic anti-desmoglein autoimmunity [7,21,36]. This interpretation appears particularly plausible in genetically predisposed individuals or in patients harboring subclinical autoreactivity prior to vaccination [14,21]. Vaccination induces a coordinated activation of innate and adaptive immune responses, and, in susceptible individuals, this transient immune stimulation could theoretically lower the threshold for clinically overt autoimmunity [21,34,36]. Proposed mechanisms include bystander activation, molecular mimicry, epitope spreading, polyclonal lymphocyte activation, and innate immune stimulation mediated by vaccine adjuvants or platform-specific immune responses [20,21,22,36]. In the context of pemphigus, these mechanisms might promote the expansion of pre-existing autoreactive lymphocyte populations and enhance the production of pathogenic anti-Dsg1 and/or anti-Dsg3 antibodies, thereby unmasking previously silent disease [7,9,21]. The relatively short latency observed in our cases may therefore support an “unmasking” hypothesis rather than the generation of an entirely new autoimmune response [9,21,36]. These observed differences in latency should be interpreted strictly as hypothesis-generating, as they may be influenced by reporting practices, distinct exposure timelines, and missing data in primary reports. Nevertheless, direct mechanistic evidence supporting these hypotheses in pemphigus remains limited [7,9]. Most published reports lack baseline serological data, longitudinal assessment of anti-desmoglein autoantibody titers, HLA characterization, or detailed immunophenotyping, thereby precluding definitive conclusions regarding pathogenesis [7,9,26].

The potential relationship between SARS-CoV-2 vaccination and pemphigus should also be considered within the broader context of post-vaccination autoimmune phenomena [21,34,37]. A spectrum of new-onset autoimmune and immune-mediated conditions has been reported in temporal association with COVID-19 vaccination, involving neurological, hematological, rheumatological, endocrine, and dermatological manifestations [21,37,38]. These observations have raised the hypothesis that the intense but transient activation of innate and adaptive immunity following vaccination may, in rare susceptible individuals, facilitate the emergence or unmasking of clinically overt autoimmunity [21,36,37]. In this context, pemphigus may represent one of several uncommon autoimmune manifestations reported after vaccination rather than a phenomenon unique to desmosomal autoimmunity [7,21,38]. Importantly, however, the accumulation of temporally associated reports does not establish a generalized propensity of SARS-CoV-2 vaccines to cause autoimmune disease, and these observations should primarily be interpreted as supporting biological plausibility and the need for continued pharmacovigilance rather than causality [21,34,39].

An additional consideration is the substantial influence of reporting and surveillance bias [34,39]. The introduction of novel vaccines, particularly during the COVID-19 pandemic, was accompanied by unprecedented pharmacovigilance and a heightened propensity to publish rare temporal associations [34,39]. Consequently, the growing number of published cases should not be interpreted as evidence of an increased biological propensity of vaccines to induce pemphigus [21,39]. Given the exceptional rarity of the disease relative to the billions of vaccine doses administered worldwide, coincidental onset remains a plausible explanation for at least a proportion of reported cases [39,40]. Overall, our findings support the possibility that vaccination may act as a trigger in selected predisposed individuals but do not support a generalized causal relationship or modify the well-established favorable benefit-risk profile of vaccination [7,9,21,39].

The comparison between drug- and vaccine-associated pemphigus represents one of the most clinically relevant findings of the present review [4,5,7,9]. Although both entities share substantial overlap in clinical presentation and therapeutic management, important differences emerged regarding the characteristics of the triggering exposure and the strength of evidence supporting a causal association [4,7,32]. Drug-associated cases were characterized by greater heterogeneity in the implicated agents, broader clinical variability, and a longer latency between exposure and disease onset [4,5,27]. In contrast, vaccine-associated cases displayed a shorter latency, were predominantly reported following SARS-CoV-2 vaccination, and were generally managed with conventional pemphigus-directed therapies [7,9,21]. These distinct temporal patterns may reflect different immunological mechanisms ultimately converging toward a common autoimmune phenotype [4,7,19,21].

From a causality perspective, drug-associated cases generally provide stronger clinical evidence for a trigger-related mechanism because several elements of causality assessment—including compatible latency, biological plausibility, and, most importantly, clinical improvement following withdrawal of the suspected agent—can be evaluated [5,32,33]. Algorithms such as the Naranjo adverse drug reaction probability scale or the World Health Organization–Uppsala Monitoring Centre (WHO-UMC) system provide structured criteria for drug causality [33,41]. In contrast, the interpretation of vaccine-associated cases is inherently more challenging because de-challenge is usually not applicable, rechallenge is rarely performed for ethical reasons, and temporal association alone cannot reliably distinguish true trigger-induced disease from coincidental onset [32,39,42]. Consequently, the level of evidence supporting individual trigger-associated cases should be considered heterogeneous rather than uniform, underscoring the need for standardized approaches to causality assessment and cautious interpretation of published reports [5,7,32,42].

From a practical perspective, clinicians should routinely investigate recent medication exposure and vaccination history in all patients presenting with newly diagnosed pemphigus, particularly when disease onset follows the introduction of an immune-modulating therapy or occurs within a temporally compatible interval after vaccination [1,4,7]. Nevertheless, attribution of causality should always rely on a comprehensive clinical assessment integrating temporal relationship, biological plausibility, exclusion of alternative explanations, disease evolution, and, whenever applicable, response to withdrawal of the suspected drug [32,33,41]. In suspected drug-associated cases, discontinuation or substitution of the offending agent should be considered whenever clinically feasible and undertaken in collaboration with the treating physician [4,5,27]. Conversely, decisions regarding subsequent vaccine doses should be individualized according to disease severity, risk of relapse, patient comorbidities, and the expected benefits of continued immunization, recognizing that the currently available evidence does not support withholding vaccination in the absence of a compelling clinical indication [1,7,21,39].

This review also highlights several important limitations of the currently available literature [1,10]. First, the systematic search relied primarily on PubMed/MEDLINE and was restricted to English-language publications, which may have inadvertently omitted some relevant cases indexed in other regional databases or non-English journals. Most published evidence consists of isolated case reports and small case series, with substantial heterogeneity in case definition, diagnostic work-up, immunological characterization, therapeutic management, and duration of follow-up [5,7,10]. Reporting of key variables—including latency, desmoglein antibody profile, direct and indirect immunofluorescence findings, treatment response, and long-term outcome—was frequently incomplete, limiting meaningful comparisons across studies [5,7]. Moreover, few reports provided sufficient information to permit a structured assessment of causality, while rechallenge data were almost universally unavailable and de-challenge could only be evaluated in drug-associated cases [32,33,41]. Collectively, these limitations substantially reduce the strength of the available evidence and preclude reliable estimates of incidence, relative risk, or attributable risk [10,39,40]. In addition, publication and reporting bias are likely to have influenced the published literature, as unusual, severe, or temporally compelling cases are inherently more likely to be reported than mild or clinically unremarkable presentations [34,39]. Finally, despite careful screening, overlap between primary reports and previously published systematic reviews, particularly in the setting of SARS-CoV-2 vaccination, cannot be completely excluded [7,9,10].

Future research should move beyond the publication of isolated case reports and prioritize standardized reporting of trigger-associated pemphigus [7,10,26]. At a minimum, future publications should systematically document the timing of exposure, the specific drug or vaccine administered, dose and treatment duration where applicable, clinical phenotype, histopathological findings, direct and indirect immunofluorescence results, anti-Dsg1 and anti-Dsg3 antibody titers, therapeutic interventions, clinical course, and long-term follow-up [1,12,26]. Furthermore, multicenter registries, prospective pharmacovigilance studies, and large observational cohorts will be essential to determine whether individual drugs or vaccines truly increase the risk of pemphigus beyond the expected background incidence [39,40,42]. Parallel mechanistic investigations integrating HLA background, autoantibody profiling, B-cell and T-cell phenotyping, and longitudinal immune monitoring may ultimately clarify why only a very small subset of exposed individuals develops disease despite the widespread use of the implicated agents [14,19,26].

While drug-associated cases often provide stronger clinical evidence through compatible latency and improvement following withdrawal of the suspected agent, interpretation of vaccine-associated cases requires greater caution because de-challenge is generally not feasible and temporal associations are expected in the context of widespread population-wide immunization [32,39,41,42]. These findings highlight the importance of clinical vigilance among dermatologists and primary care physicians rather than restricting vaccination [21,39,40]. Clinicians should remain aware of potential temporal associations to ensure prompt diagnostic recognition and management should cutaneous symptoms arise following immunization [21,39,40,42]. Such balanced communication may be particularly relevant in individuals with a previous history of autoimmune blistering disease and may facilitate prompt recognition of new mucocutaneous manifestations occurring after vaccination [1,7,21].

## 6. Conclusions

This systematic review delineates the reported clinical spectrum of new-onset pemphigus temporally associated with pharmacological agents and vaccines. Drug-associated cases demonstrate substantial etiological diversity, longer median latency, and frequent clinical improvement upon drug withdrawal. In contrast, vaccine-associated cases present with a shorter median latency—predominantly following SARS-CoV-2 immunization—a descriptive pattern that may reflect transient immune unmasking in predisposed hosts. Given that population denominators are unavailable from case reports, these temporal associations remain descriptive. Clinicians should routinely investigate recent exposures in newly diagnosed patients, while standardized prospective registries are needed to further clarify these associations.

## Figures and Tables

**Table 1:** Overall methodological quality assessment of included primary case reports using the Joanna Briggs Institute (JBI) critical appraisal checklist (*N* = 42 published studies).

JBI Critical Appraisal Domain	Yes (*n*, %)	No (*n*, %)	Unclear (*n*, %)	Compliance Level
Q1. Patient demographic characteristics clearly described	40 (95.2%)	0 (0.0%)	2 (4.8%)	High
Q2. Patient history clearly presented as a timeline	38 (90.5%)	1 (2.4%)	3 (7.1%)	High
Q3. Current clinical condition clearly described	41 (97.6%)	0 (0.0%)	1 (2.4%)	High
Q4. Diagnostic tests and results clearly described	42 (100.0%)	0 (0.0%)	0 (0.0%)	Optimal
Q5. Intervention/treatment procedure clearly described	39 (92.9%)	1 (2.4%)	2 (4.8%)	High
Q6. Post-intervention clinical outcome clearly described	37 (88.1%)	2 (4.8%)	3 (7.1%)	High
Q7. Adverse events or unanticipated events identified	33 (78.6%)	4 (9.5%)	5 (11.9%)	Moderate
Q8. Case report provides clear takeaway lessons	41 (97.6%)	0 (0.0%)	1 (2.4%)	High

## Data Availability

The data that support the findings of this study are available from the corresponding author upon reasonable request.

## Supplementary Materials

The following supporting information can be downloaded at: https://www.mdpi.com/article/10.3390/vaccines14090828/s1, File S1: PRISMA 2020 checklist.
